# Use of Nonrecommended Antivirals Among Medicare Beneficiaries With HIV

**DOI:** 10.1001/jamanetworkopen.2025.8296

**Published:** 2025-05-01

**Authors:** Jose F. Figueroa, Dannie Dai, Florence Ebem, Ciara Duggan, Jacqueline Chu, Grace Luu, Jessica Phelan, Rajesh T. Gandhi, E. John Orav, Emily P. Hyle

**Affiliations:** 1 Department of Health Policy and Management, Harvard T.H. Chan School of Public Health, Boston, Massachusetts; 2 Department of Medicine, Brigham and Women’s Hospital, Boston, Massachusetts; 3 Medical Practice Evaluation Center, Department of Medicine, Massachusetts General Hospital, Boston; 4 Division of Infectious Diseases, Department of Medicine, Massachusetts General Hospital, Boston

## Abstract

This cross-sectional study examines contemporary evidence on the extent to which Medicare beneficiaries with HIV fill older, more toxic antivirals as part of their antiretroviral therapy.

## Introduction

Antiretroviral therapy (ART) for people with HIV has improved substantially, becoming more effective and less toxic and improving quality of life and life expectancy.^1,2^ Some people with HIV take older, more toxic antivirals, despite clinical guidelines recommending discontinuation in 2017.^2^ Barriers that may delay the transition from older regimens may include patient preference, costs associated with newer antivirals, and clinician factors.^3^ These concerns may be particularly relevant when treating Medicare beneficiaries with HIV who may have multimorbidities and multiple prescribed medications.^4,5^ Contemporary evidence is lacking on the extent to which Medicare beneficiaries with HIV fill older, more toxic antivirals as part of their ART regimen.

## Methods

This cross-sectional study used a 20% random sample of traditional Medicare beneficiaries with Part D coverage from 2013 to 2021. The study was approved by the Harvard Longwood Campus Institutional Review Board, which waived informed consent as data were deidentified. We followed the STROBE reporting guideline.

For each year, we identified filled antiviral prescriptions for beneficiaries with HIV. Expert clinicians (J.C., E.P.H.) reviewed prescribed antivirals and categorized them as nonrecommended based on drug toxicity and whether more effective alternatives were available. The remaining antivirals were classified as preferred. Further beneficiary and classification details are provided in the eMethods in Supplement 1.

We obtained the following person-year–level characteristics: demographics (age, sex, and race and ethnicity), Medicaid dual eligibility, Part D Low-Income Subsidy eligibility, and other chronic conditions. Race and ethnicity were categorized using the Research Triangle Institute variable, which applies an algorithm on self-reported data, and included given known disparities in HIV treatment. We compared characteristics of beneficiaries with HIV with at least 1 nonrecommended antiviral vs only preferred antivirals using standardized mean differences (SMDs), with values less than 0.10 not considered meaningful. We then assessed trends over time by calculating the proportion of beneficiaries with HIV with at least 1 nonrecommended vs only preferred antivirals. Data were analyzed from June through November 2024 using R, version 4.2.1 (R Foundation).

## Results

A total of 1052 beneficiaries with HIV contributing 2901 person-years (2151 aged <65 years [74.1%], 728 female [25.1%] and 2173 male [74.9%]) filled at least 1 nonrecommended antiviral prescription, and 28 019 contributing 135 791 person-years (75.9% aged <65 years, 25.6% female and 74.4% male) filled only preferred antiviral prescriptions. Beneficiaries taking nonrecommended antivirals were more likely to live in the South (50.5% vs 45.6%; SMD, 0.10) and less likely to live in the West (13.4% vs 18.4%; SMD, 0.14) (Table). There were no meaningful differences in characteristics between beneficiaries taking nonrecommended vs preferred antivirals regarding age, sex, race and ethnicity, Medicaid dual eligibility, or Part D Low-Income Subsidy eligibility. The most used nonrecommended antivirals were didanosine (797 person-years [27.5%]) and nelfinavir (733 person-years [25.3%]). The proportion of beneficiaries with HIV who received at least 1 nonrecommended antiviral decreased from 5.1% in 2013 to 0.1% in 2021 (Figure).

**Table.** Characteristics of Medicare Beneficiaries With HIV Taking Nonrecommended vs Preferred Antivirals, 2013-2021

Characteristic	Person-years, No. (%)		SMD
	Nonrecommended antivirals	Preferred antivirals	
No. of beneficiaries	1052	28 019	NA
No. of person-years contributed	2901	135 791	NA
Age group			
<65 y	2151 (74.1)	103 076 (75.9)	0.04
≥65 y	750 (25.9)	32 715 (24.1)	
Sex			
Female	728 (25.1)	34 762 (25.6)	0.01
Male	2173 (74.9)	101 029 (74.4)	
Race and ethnicity			
Black	1111 (38.3)	55 140 (40.6)	0.05
Hispanic	315 (10.9)	16 878 (12.4)	0.05
White	1417 (48.8)	60 743 (44.7)	0.08
Other^a^	58 (2.0)	3030 (2.2)	0.02
Medicaid dual eligibility	1915 (66.0)	91 546 (67.4)	0.03
Part D Low-Income Subsidy eligibility	2355 (81.2)	110 286 (81.2)	0.00
Chronic conditions			
Alzheimer disease or related dementia	177 (6.1)	8862 (6.5)	0.02
Chronic kidney disease	706 (24.3)	40 390 (29.7)	0.12
Chronic obstructive pulmonary disease	346 (11.9)	17 809 (13.1)	0.04
Congestive heart failure	371 (12.8)	15 033 (11.1)	0.05
Diabetes	808 (27.9)	33 803 (24.9)	0.07
Ischemic heart disease	703 (24.2)	28 810 (21.2)	0.07
Cancer	158 (5.4)	6779 (5.0)	0.02
Depressive disorder	942 (32.5)	53 307 (39.3)	0.14
Bipolar disorders	227 (7.8)	15 948 (11.7)	0.13
Schizophrenia or psychotic disease	177 (6.1)	10 105 (7.4)	0.05
Rurality			
Rural	361 (12.4)	14 663 (10.8)	0.06
Urban	2540 (87.6)	121 128 (89.2)	
US region			
Midwest	344 (11.9)	18 057 (13.3)	0.04
Northeast	705 (24.4)	30 870 (22.7)	0.04
South	1464 (50.5)	61 925 (45.6)	0.10
West	388 (13.4)	24 939 (18.4)	0.14
Nonrecommended antivirals^b^			
Didanosine	797 (27.5)	NA	NA
Nelfinavir	733 (25.3)	NA	NA
Stavudine	644 (22.2)	NA	NA
Saquinavir	445 (15.3)	NA	NA
Abacavir, lamivudine, and zidovudine^c^	302 (10.4)	NA	NA
Indinavir	155 (5.3)	NA	NA

Abbreviations: NA, not applicable; SMD, standardized mean difference.

^a^ Other encapsulates the following racial and ethnic groups: American Indian or Alaska Native, Asian, Native Hawaiian or Pacific Islander, other, and unknown.

^b^ Some people were prescribed more than 1 nonrecommended antiviral medication within a given year, so the percentages do not sum to 100%.

^c^ Considered nonrecommended only if given alone without other antivirals.

**Figure.**
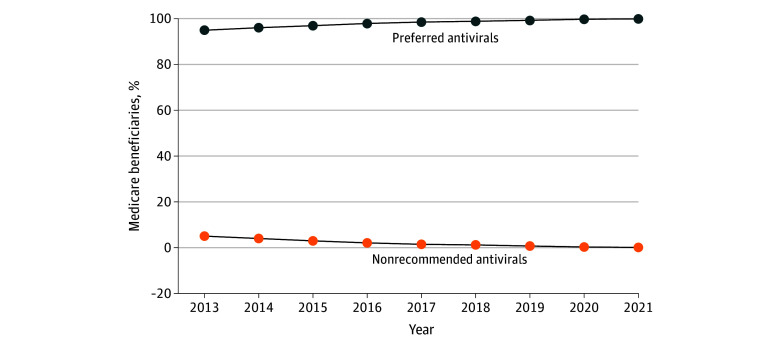
Proportion of Medicare Beneficiaries With HIV Taking Nonrecommended vs Preferred Antivirals, 2013-2021

## Discussion

Research into care quality for the aging population with HIV is crucial as more than 50% are now aged 50 years or older. In this cross-sectional study of Medicare beneficiaries with HIV, the proportion who filled nonrecommended antiviral prescriptions decreased substantially from 5.1% in 2013 to only 0.1% in 2021 and without a sharp change in 2017. Overall, these results suggest that clinicians have been discontinuing use of older toxic antivirals over time. However, this analysis raises concerns that beneficiaries living in the South had been receiving nonrecommended antivirals. Further research should assess how many beneficiaries switched to less toxic antivirals over time (rather than being censored), characteristics associated with switching to safer alternatives, alternative antivirals prescribed instead, and how more recent ART guideline recommendations may influence future prescription patterns.^6^

The study was limited to traditional Medicare beneficiaries with HIV and may not be generalizable to people with HIV with commercial insurance, Medicaid, or Medicare Advantage. HIV diagnoses depended on claims data, and inclusion in the study cohort required filling an antiviral prescription.

## Notes

Supplement 1.
eMethods.

eReferences.

Supplement 2.
Data Sharing Statement
