# Supplementary material for: Use of Nonrecommended Antivirals Among Medicare Beneficiaries With HIV
Source: JAMA Netw Open. 2025 May 1;8(5):e258296. doi: 10.1001/jamanetworkopen.2025.8296 (PMC12046424; doi:10.1001/jamanetworkopen.2025.8296)
Supplement: Supplement 1. — eMethods. eReferences. [file jamanetwopen-e258296-s001.pdf]

Supplemental Online Content

Figueroa JF, Dai D, Ebem F, et al. Use of nonrecommended antivirals among Medicare beneficiaries with HIV. *JAMA Netw Open*. 2025;8(5):e258296. doi:10.1001/jamanetworkopen.2025.8296

eMethods

eReferences

This supplemental material has been provided by the authors to give readers additional information about their work.

© 2025 Figueroa JF et al. *JAMA Network Open*.

eMethods

Identification of People with HIV and Other Chronic Conditions

Individuals with a known HIV diagnosis were identified using the Chronic Conditions Warehouse (CCW) algorithm.[1] The CCW algorithms were also used to identify the other chronic conditions. The CCW algorithm was created by the Centers for Medicare and Medicaid Services (CMS) to increase the sensitivity and specificity of individuals actually having each chronic condition when based on administrative claims data.

Identification of "Not Recommended" Antivirals

Two clinicians (JC, EPH) independently reviewed the list of prescribed antivirals and identified medications that should no longer have been recommended during the study period (2013-2021) given higher toxicity profile and availability of more effective alternatives. The categorization of these "not recommended" antivirals had 100% consensus between the two clinicians. The remaining antivirals were designated as "preferred" antivirals. The National Drug Code Directory data schema was used to determine the antivirals used to treat HIV.[2]

DHHS Guidelines Recommendations for Discontinuation of Antivirals in Regimens Prescribed to Adults and Adolescents with HIV in the US

Starting in 2003, DHHS Guidelines first included recommendations on antivirals to stop if in regimens being prescribed to people on ART in the US (i.e., in contrast to preferred antiviral regimens for initiation of ART in people with HIV).[3] However, the antivirals on this list (2013-17) were mostly combinations with high toxicity or single drug regimens. Starting in 2017, the DHHS guidelines added specific antivirals that were recommended to discontinue if they were prescribed as part of an existing regimen.[4]

Determination of Patient-Level Demographics

Person-year-level characteristics were obtained from the Medicare Beneficiary Summary File, including race and ethnicity. Race and ethnicity were included in the analysis to assess for racial inequities in the quality of care for older PWH. The authors performed this analysis given historical and current racial inequities in access to HIV treatment and quality of care among PWH.

Race and ethnicity were defined using the Research Triangle Institute (RTI) race code variable available in administrative claims data as Black, Hispanic, White, or Other. To be compliant with the CMS small cell size suppression policy, we grouped American Indian/Alaska Native, Asian/Pacific Islander, Other or Unknown into the Other category. The RTI race code is determined from multiple records, including the Social Security Administration records, addresses, and names. The RTI race variable improves classification of Hispanics and Asians/Pacific Islanders by applying an imputation

© 2025 Figueroa JF et al. *JAMA Network Open*.

algorithm that uses names from the US Census and geography. The limitations on the accuracy of racial and ethnic classifications in CMS administrative data is well-established.[5]

eReferences

1. Centers for Medicare and Medicaid. Chronic Conditions Data Warehouse. Other Chronic Health, Mental Health, and Potentially Disabiling Conditions. Accessed December 5, 2024. https://www2.ccwdata.org/web/guest/condition-categories-other

2. National Bureau of Economic Research (NBER). National Drug Code. NBER. Accessed December 5, 2024. https://www.nber.org/research/data/national-drug-code

3. Panel on Antiretroviral Guidelines for Adults and Adolescents. Guidelines for the Use of Antiretroviral Agents in HIV-1-infected Adults and Adolescents. 2003. Department of Health and Human Services. Accessed February 24, 2025. https://clinicalinfo.hiv.gov/sites/default/files/guidelines/archive/AdultandAdolescentGL06142003005.pdf

4. Panel on Antiretroviral Guidelines for Adults and Adolescents. Guidelines for the Use of Antiretroviral Agents in Adults and Adolescents Living with HIV. 2017. Department of Health and Human Services. Accessed February 24, 2025. https://clinicalinfo.hiv.gov/sites/default/files/guidelines/archive/AdultandAdolescentGL003510.pdf

5. Jarrín OF, Nyandege AN, Grafova IB, Dong X, Lin H. Validity of Race and Ethnicity Codes in Medicare Administrative Data Compared With Gold-standard Self-reported Race Collected During Routine Home Health Care Visits. *Med Care*. 2020;58(1):e1-e8. doi:10.1097/MLR.0000000000001216

© 2025 Figueroa JF et al. *JAMA Network Open*.
