# Supplementary material for: Use of Nonrecommended Antivirals Among Medicare Beneficiaries With HIV
Source: JAMA Netw Open. 2025 May 1;8(5):e258296. doi: 10.1001/jamanetworkopen.2025.8296 (PMC12046424; doi:10.1001/jamanetworkopen.2025.8296)
Supplement: Supplement 2. — Data Sharing Statement [file jamanetwopen-e258296-s002.pdf]

Data Sharing Statement

Figueroa. Use of Nonrecommended Antivirals Among Medicare Beneficiaries With HIV. *JAMA Netw Open*. Published May 01, 2025. doi:10.1001/jamanetworkopen.2025.8296

Data
Data available: No

Additional Information
Explanation for why data not available: These data cannot be shared as part of our CMS data use agreement. However, these data are available for research use through ResDAC if one wishes to pursue their own data use agreement.
